# Association of the Risk of Childhood Asthma at Age 6 With Maternal Allergic or Immune-Mediated Inflammatory Diseases: A Nationwide Population-Based Study

**DOI:** 10.3389/fmed.2021.713262

**Published:** 2021-08-02

**Authors:** Deng-Ho Yang, Chun-Shih Chin, Wen-Cheng Chao, Ching-Heng Lin, Yun-Wen Chen, Yi-Hsing Chen, Hsin-Hua Chen

**Affiliations:** ^1^Division of Rheumatology/Immunology/Allergy, Department of Internal Medicine, Taichung Armed Forces General Hospital, Taichung, Taiwan; ^2^Department of Medical Laboratory Science and Biotechnology, Central Taiwan University of Science and Technology, Taichung, Taiwan; ^3^Division of Rheumatology/Immunology/Allergy, Department of Internal Medicine, Tri-Service General Hospital, Taipei, Taiwan; ^4^Division of Pulmonary and Critical Care Medicine, Department of Internal Medicine, Hyperbaric Oxygen Therapy Center, Taichung Veterans General Hospital, Taichung, Taiwan; ^5^Division of Chest Medicine, Department of Internal Medicine, Taichung Veterans General Hospital, Taichung, Taiwan; ^6^Department of Computer Science, Tunghai University, Taichung, Taiwan; ^7^Department of Critical Care Medicine, Taichung Veterans General Hospital, Taichung, Taiwan; ^8^Department of Industrial Engineering and Enterprise Information, Tunghai University, Taichung, Taiwan; ^9^Department of Medical Research, Taichung Veterans General Hospital, Taichung, Taiwan; ^10^Department of Healthcare Management, National Taipei University of Nursing and Health Sciences, Taipei, Taiwan; ^11^Department of Public Health, College of Medicine, Fu Jen Catholic University, New Taipei City, Taiwan; ^12^Division of Allergy, Immunology, and Rheumatology, Department of Internal Medicine, Taichung Veterans General Hospital, Taichung, Taiwan; ^13^School of Medicine, National Yang-Ming University, Taipei, Taiwan; ^14^Division of General Internal Medicine, Department of Internal Medicine, Taichung Veterans General Hospital, Taichung, Taiwan; ^15^Institute of Biomedical Science and Rong-Hsing Research Center for Translational Medicine, Chung-Hsing University, Taichung, Taiwan; ^16^Institute of Public Health and Community Medicine Research Center, National Yang-Ming University, Taipei, Taiwan

**Keywords:** childhood, asthma, immune medicated inflammatory disease, incidence, risk, maternal

## Abstract

**Objective:** This study aimed to assess the associations of the risk of asthma diagnosed in children aged 6 years or younger and having maternal immune-mediated inflammatory diseases (IMIDs), including systemic lupus erythematosus (SLE), systemic sclerosis (SSc), inflammatory myositis, rheumatoid arthritis (RA), Sjögren's syndrome (SS), ankylosing spondylitis (AS), and autoimmune thyroiditis.

**Methods:** A total of 628,878 singleton newborns documented in 2006–2009 and followed up for at least 6 years were identified. Overall, 153,085 (24.3%) children developed asthma at the age of ≤ 6 years. Two groups of maternal ages, i.e., <35 and ≥35 years, were evaluated. The associations of the risk of asthma occurring in children who were 6 years old or younger and had maternal IMIDs were examined.

**Results:** The risk of asthma increased in children whose mothers had SLE [odds ratio (OR), 1.13; 95% confidence intervals (CI), 1.00–1.27; *p* = 0.04), RA (OR, 1.21; 95% CI, 1.07–1.38; *p* = 0.003), inflammatory myositis (OR, 1.41; 95% CI, 1.12–1.74; *p* = 0.003), asthma (OR, 1.58; 95% CI, 1.52–1.63), allergic rhinitis (OR, 1.30; 95% CI, 1.28–1.32), or atopic dermatitis (OR, 1.07; 95% CI, 1.02–1.12). Conversely, this increased risk was not observed in children whose mothers had AS (OR, 1.02; 95% CI, 0.87–1.20), SS (OR, 0.96; 95% CI, 0.86–1.07), SSc (OR, 1.28; 95% CI, 0.77–2.14), or autoimmune thyroiditis (OR, 1.01; 95% CI, 0.95–1.07). Other risk factors of childhood asthma included high urbanization level, preterm birth, and low birth weight.

**Conclusion:** The risk of childhood asthma at 6 years of age increased in children whose mothers suffered from SLE, RA, inflammatory myositis, asthma, allergic rhinitis, and atopic dermatitis.

## Introduction

Asthma is a common chronic inflammatory respiratory disease in children, and its prevalence has increased (1). In the mechanism of asthma, persistent airway inflammation with hyper-responsiveness and different airway remodeling processes can occur. Childhood asthma causes varying considerable burdens on affected children and their families (2, 3). Its well-established risk factors are parental asthma, prenatal environmental tobacco smoke exposure, and prematurity (4).

Immune-mediated inflammatory diseases (IMIDs) include a group of diseases that alter immune regulation, thereby, causing chronic inflammation in single or multiple organs. Type 2 inflammation contributes to the development of allergic diseases, including asthma, urticaria, and atopic dermatitis. In the early stage of asthma, epithelial cytokines and chemokines are produced when epithelial cells interact with viruses and bacteria (5). Around 3–7% of the population with an estimated incidence of 80 per 105 person-years is affected by IMIDs worldwide (6–8). Despite the links between maternal diseases and childhood health status, evidence on the association between maternal IMIDs and childhood asthma is limited. The risk of asthma in children is positively correlated with maternal systemic lupus erythematosus (SLE) (9). Couture et al. found that the risk of allergic conditions, including asthma, is higher in children whose mothers have SLE than in children born to the general population in Canada (10).

Asthma or other allergy-related diseases are associated with genetic and environmental factors. In the development of asthma, epigenetic regulators are one of the factors of the differentiation and plasticity of T-helper cells through epigenetic histone and DNA modification (11). Occupational asthma, aspirin-exacerbated respiratory disease, tobacco smoke-related airway dysfunction, and farm-related atopic diseases are related to gene–environment interaction, and one of its mechanisms is epigenetics (12). The risk of childhood asthma is associated with the maternal, paternal, and postnatal environmental modulation of allergic responses by different epigenetic mechanisms; however, maternal atopy affects pre- and postnatal allergic cytokine production (13). Our study focused on the evaluation of maternal conditions.

Advanced maternal age is usually defined as being 35 years or older, which is believed to predispose mothers to numerous adverse outcomes during pregnancy (14). Metsala et al. used different age deliveries (<25, 25–29, 30–34, and ≥35 years) to evaluate the risk of childhood asthma (15). In the present study, the age of 35 years was set as the cutoff of maternal age groups. However, to our knowledge, studies have yet to concurrently investigate the associations between various maternal IMIDs and the risk of childhood asthma.

This study aimed to assess the relationship of the risks of childhood asthma at the age of 6 years with various maternal allergic diseases and maternal IMIDs. Allergic diseases included asthma, allergic rhinitis, and atopic dermatitis. IMIDs were SLE, rheumatoid arthritis (RA), Sjögren's syndrome (SS), systemic sclerosis (SSc), inflammatory myositis, ankylosing spondylitis (AS), and autoimmune thyroiditis, including Grave's disease and Hashimoto's thyroiditis.

## Methods

### Ethics

This study was approved by the Institutional Review Board of Taichung Veterans General Hospital (number CE19057B). Personal information was anonymized before data analyses; hence, the requirement for informed consent was waived.

### Study Design

A retrospective matched case-control study was performed.

### Data Source

Several government-held databases were used: 2003–2015 National Health Insurance Research Database (NHIRD), 2003–2015 Taiwan Birth Registry Database (TBRD), and 2004–2014 Taiwan Maternal and Child Health Database (TMCHD). The NHIRD currently covers over 99% of the Taiwanese residents with comprehensive records of the following data: enrollment registry date, insurance details, dates of outpatient and services, diagnostic codes, medication prescription, medical expenditures, examination items, and surgical procedures. However, it does not provide the following personal data: tobacco use, alcohol drinking, body weight, and body length. The Bureau of National Health Insurance routinely checks original medical records to improve the accuracy of the data in the NHIRD (16). In Taiwan, live births and stillbirths (weighted >500 mg or older than 20 weeks) are registered in the TBR within 15 days. The TBRD provides detailed information of newborns (e.g., birthplace, birth date, sex, gestational age, birth weight, single or multiple births, birth order, and Apgar score) and mothers (e.g., age at delivery, residential address, and pregnancy complications). The TMCHD contains encrypted personal identification numbers of newborns and their parents in a nationwide birth cohort in 2004–2014. Researchers can request the interlinking of all information of children and their mothers in the NHIRD and the TBRD from the Taiwan Health and Welfare Data Science Center of the Ministry of Health and Welfare by using the encrypted personal identification numbers from the TMCHD.

**Figure 1:**
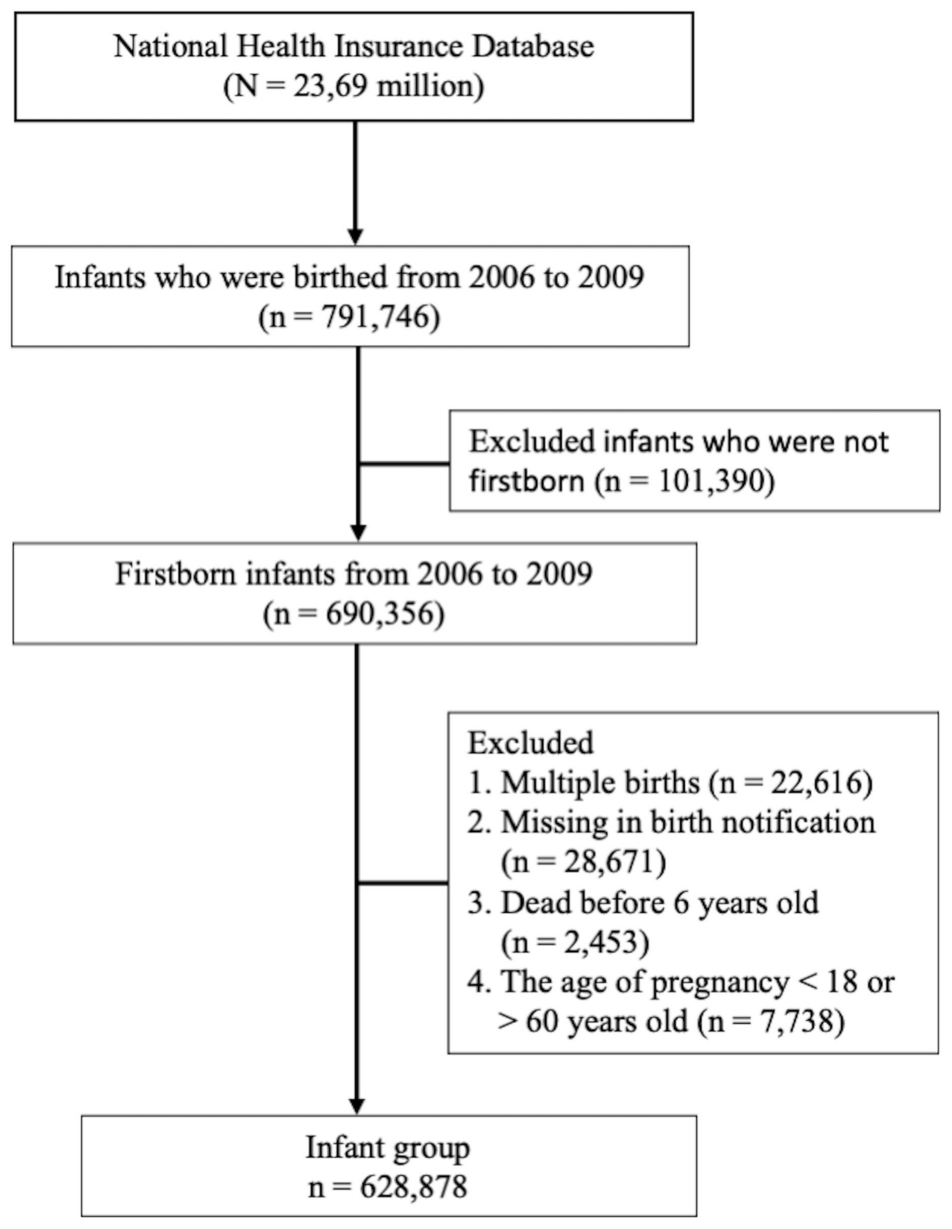
Flowchart of the study subject enrollment.

Children were considered to have childhood asthma at the age of 6 years if they were diagnosed with asthma during at least three outpatient visits (International Classification of Diseases, Ninth Revision, Clinical Modification ICD-9-CM code 493). Mothers were considered to have allergic diseases if they had at least three ambulatory visits or one inpatient visit with a diagnosis of asthma (ICD-9-CM code 493), allergic rhinitis (ICD-9-CM code 477), or atopic dermatitis (ICD-9-CM code 691). Mothers were also considered to have IMIDs if they had at least three ambulatory visits or one inpatient visit with a diagnosis of AS (ICD-9-CM 720.0), autoimmune thyroiditis (Graves' disease, ICD-9-CM code 242.0; or Hashimoto's thyroiditis, ICD-9-CM code 245.2), SLE (ICD-9-CM code 710.0), SSc (ICD-9-CM code 710.1), SS (ICD-9-CM code 710.2), inflammatory myositis (dermatomyositis, ICD-9-CM code 710.3; polymyositis, ICD-9-CM code 710.4), or RA (ICD-9-CM code 714.0) before they gave birth. A total of 628,878 infants were included after the following data were excluded: multiple births, missing birth notification, survival under 6 years old, and pregnancy age of <18 or ≥60 years. Mothers aged <18 years were excluded because the Institutional Review Board approved only the inclusion of “adult mothers” in the present study. Pregnant mothers aged >60 years were also excluded because such condition was assumed a mistake given that pregnancy beyond the age of 60 years is almost impossible. The flow chart of study subject enrollment is shown in Figure 1.

### Statistical Analysis

Continuous variables were presented as mean ± standard deviation, and categorical variables were presented as the percentage of patients. Differences in continuous and categorical variables were examined using Student's *t*-test and Pearson's χ^2^ test, respectively. The associations between variables and the risks of childhood asthma were determined by estimating odds ratios (ORs) with 95% confidence intervals (CIs) *via* multivariable logistic regression analysis after adjustments for potential confounders were made. Data were statistically analyzed with SAS version 9.3 (SAS Institute, Inc., Cary, NC, USA). Results with two-tailed *p* < 0.05 were considered statistically significant.

## Results

### Study Population

Figure 1 shows the flowchart of the study subject enrollment. A total of 791,746 infants were delivered between January 1, 2006, and December 31, 2009. Of these infants, 690,356 were firstborn. Infants with the following characteristics were excluded: those who were part of multiple childbirths, had missing birth notifications, were dead before 4 years old, and had mothers who were <18 or >60 years during pregnancy. Afterward, 628,878 children were included in the study: 300,290 females (47.7%) and 328,588 (52.3%) males. Among these children, 153,085 developed asthma (24.3%), and the place of residence was highly urbanized. The average age at maternal pregnancy was 29.1 ± 4.59 years, and the weeks of pregnancy were 38.4 ± 1.52. In this study, the utilization levels were categorized on the basis of the mean values of standardized scores of the following variables: population density (persons/km^2^), percentage of people with educational levels of college or above, percentage of elderly people over 65 years old, percentage of agricultural workers, and number of physicians per 100,000 people (17). Urbanization levels were stratified into seven clusters ranging from level 1 (most urbanized) to level 7 (least urbanized). The detailed definitions of utilization levels are shown in Supplementary Table 1. However, these four levels were combined into one level (level 4) because both cohorts had a small number of patients in levels 4 through 7. The following autoimmune-related comorbidities were found in the mothers: asthma (2.24%), allergic rhinitis (12.5%), atopic dermatitis (1.74%), SLE (0.22%), RA (0.19%), SS (0.28%), SSc (0.01%), inflammatory myositis (0.06%), autoimmune thyroiditis (0.97%), and AS (0.13%). The general data of the child and maternal status are presented in Table 1.

**Table 1:** Demographic data of study subjects.

**Variable**	***n* = 628,878**
**Child status**	
Sex	
Female	300,290 (47.7)
Male	328,588 (52.3)
Urbanization levels	
1 (highest)	191,780 (30.5)
2	201,557 (32.1)
3	114,312 (18.2)
4+ (lowest)	121,229 (19.3)
Asthma	153,085 (24.3)
Birth weight (g), mean (SD)	3,110 (427)
**Mother status**	
Parity	
1	427,514 (68.0)
2	176,644 (28.1)
3+	24,720 (3.93)
Comorbidity	
Asthma	14,087 (2.24)
AR	78,743 (12.5)
AD	10,962 (1.74)
GDM	10,196 (1.62)
Preeclampsia	4,663 (0.74)
AS	790 (0.13)
Autoimmune thyroiditis	6,108 (0.97)
SLE	1,399 (0.22)
Systemic sclerosis	70 (0.01)
Sjögren's syndrome	1,752 (0.28)
DM/PM	374 (0.06)
RA	1,188 (0.19)

*Data are shown as number (percentage) unless otherwise specified. SD, standard deviation; AR, allergic rhinitis; AD, atopic dermatitis; GDM, gestational diabetes mellitus; AS, ankylosing spondylitis; SLE, systemic lupus erythematosus; DM/PM, dermatomyositis/polymyositis; RA, rheumatoid arthritis*.

### Maternal Pregnancy Conditions and the Risk of Childhood Asthma

High urbanization levels may increase the risk of childhood asthma compared with low urbanization levels. The highest urbanization level had an OR of 1.26 with 95% CI of 1.24–1.29. Preterm birth or low birth weight had an increased risk of childhood asthma (OR, 1.21; 95% CI, 1.18–1.24; OR, 1.07; 95% CI, 1.04–1.09). When the maternal age was more than 35 years, the risk of childhood asthma decreased (OR, 0.89; 95% CI, 0.87–0.90). Therefore, the decreased risk of childhood asthma was related to high parity in mothers. The risk of childhood asthma under different maternal pregnant conditions and comorbidities is shown in Table 2.

**Table 2:** Risk of childhood asthma in different maternal pregnant conditions and comorbidities.

	**Normal children** ***n* = 475,793**	**Childhood asthma** ***n* = 153,085**	**cOR** ** (95% CI)**	**aOR** ** (95% CI)**	***p*-Value**
Child sex					
Female	236,210 (49.7)	64,080 (41.9)	1	1	-
Male	239,583 (50.3)	89,005 (58.1)	1.37 (1.35–1.39)	1.37 (1.36–1.39)	<0.0001
Urbanization levels					
1 (highest)	142,079 (29.9)	49,701 (32.5)	1.26 (1.24–1.28)	1.26 (1.24–1.29)	<0.0001
2	149,976 (31.5)	51,581 (33.7)	1.24 (1.22–1.26)	1.24 (1.22–1.26)	<0.0001
3	88,855 (18.7)	25,457 (16.6)	1.03 (1.01–1.05)	1.03 (1.01–1.05)	0.006
4+ (lowest)	94,883 (19.9)	26,346 (17.2)	1	1	-
Preterm					
No	444,970 (93.5)	140,849 (92.0)	1	1	-
Yes	30823 (6.48)	12,236 (7.99)	1.25 (1.23–1.28)	1.21 (1.18–1.24)	<0.0001
Low birth weight					
No	445,895 (93.7)	142,279 (92.9)	1	1	-
Yes	29,898 (6.28)	10,806 (7.06)	1.13 (1.11–1.16)	1.07 (1.04–1.09)	<0.0001
Pregnant age					
<35	424,406 (89.2)	137,900 (90.1)	1	1	-
≥35	51,387 (10.8)	15,185 (9.92)	0.91 (0.89–0.93)	0.89 (0.87–0.90)	<0.0001
Parity					
1	321,318 (67.5)	106,196 (69.4)	1	1	-
2	135,164 (28.4)	41,480 (27.1)	0.93 (0.92–0.94)	0.93 (0.92–0.95)	<0.0001
3+	19,311 (4.06)	5,409 (3.53)	0.85 (0.82–0.87)	0.86 (0.83–0.89)	<0.0001
Maternal comorbidity					
Asthma	9,011 (1.89)	5,076 (3.32)	1.78 (1.72–1.84)	1.58 (1.52–1.63)	<0.0001
AR	55,422 (11.7)	23,321 (15.2)	1.36 (1.34–1.39)	1.30 (1.28–1.32)	<0.0001
AD	8,057 (1.69)	2,905 (1.90)	1.12 (1.08–1.17)	1.07 (1.03–1.12)	0.002
Preeclampsia	7675 (1.61)	2,521 (1.65)	1.02 (0.98–1.07)	1.01 (0.96–1.05)	0.82
GDM	3,417 (0.72)	1,246 (0.81)	1.13 (1.06–1.21)	1.03 (0.96–1.10)	0.42
AS	586 (0.12)	204 (0.13)	1.08 (0.92–1.27)	1.02 (0.87–1.20)	0.80
Autoimmune thyroiditis	4,586 (0.96)	1,522 (0.99)	1.03 (0.97–1.09)	1.01 (0.95–1.07)	0.77
SLE	999 (0.21)	400 (0.26)	1.25 (1.11–1.40)	1.13 (1.00–1.27)	0.04
Systemic sclerosis	48 (0.01)	22 (0.01)	1.42 (0.86–2.36)	1.28 (0.77–2.14)	0.34
Sjögren's syndrome	1,306 (0.27)	446 (0.29)	1.06 (0.95–1.18)	0.96 (0.86–1.07)	0.45
DM/PM	254 (0.05)	120 (0.08)	1.47 (1.18–1.83)	1.40 (1.12–1.74)	0.003
RA	838 (0.18)	350 (0.23)	1.30 (1.15–1.47)	1.21 (1.07–1.38)	0.003

*cOR, crude odds ratio; aOR, adjusted odds ratio; AR, allergic rhinitis; AD, atopic dermatitis; GDM, gestational diabetes mellitus; AS, ankylosing spondylitis; SLE, systemic lupus erythematosus; DM/PM, dermatomyositis/polymyositis; RA, rheumatoid arthritis. The model was mutually adjusted for all variables in this table*.

### High Risk of Childhood Asthma in Maternal Comorbidities, Including Allergic Diseases, Systemic Lupus Erythematosus, Inflammatory Myositis, and Rheumatoid Arthritis

Among the different maternal IMIDs, the increased risks of childhood asthma were observed in SLE (OR,1.13; 95% CI,1.00–1.27), RA (OR, 1.21; 95% CI, 1.07–1.38), and inflammatory myositis (OR, 1.40; 95% CI, 1.12–1.74). Additionally, the risk of childhood asthma might be increased in infants whose mothers had different allergic diseases, including asthma (OR, 1.58; 95% CI, 1.52–1.63), allergic rhinitis (AR) (OR, 1.3; 95% CI, 1.28–1.32), and atopic dermatitis (AD) (OR, 1.07; 95% CI, 1.03–1.12). The risk of childhood asthma under different maternal pregnant conditions and comorbidities is presented in Table 2.

### High Risk of Childhood Asthma, Allergic Rhinitis, and a Topic Dermatitis in Mothers With Allergic Diseases

When mothers had a history of asthma, AR, or AD, the risk of childhood allergic diseases, including asthma, AR, and AD, increased (Table 3). The ORs of childhood allergic diseases were 1.52, 1.93, and 2.73 when mothers had one, two, and three allergic diseases, respectively. Therefore, the risk of childhood allergic disease increased when mothers had one, two, or three allergic diseases. The overall risk of childhood allergic diseases in mothers with one, two, or three allergic diseases is summarized in Table 4.

**Table 3:** Risk of allergic diseases of children in different maternal allergic status.

**Maternal status**	**Children allergic disease outcome**			
	**Overall**	**Asthma**	**AR**	**AD**
	**OR (95% CI)**	**OR (95% CI)**	**OR (95% CI)**	**OR (95% CI)**
Asthma				
No	1	1	1	1
Yes	1.34 (1.29–1.39)	1.58 (1.52–1.64)	1.23 (1.19–1.27)	1.25 (1.21–1.30)
AR				
No	1	1	1	1
Yes	1.55 (1.52–1.57)	1.30 (1.28–1.32)	1.61 (1.59–1.64)	1.31 (1.28–1.33)
AD				
No	1	1	1	1
Yes	1.30 (1.25–1.35)	1.08 (1.03–1.12)	1.13 (1.08–1.17)	1.51 (1.45–1.57)

*OR, odds ratio; AR, allergic rhinitis; AD, atopic dermatitis; ref, reference. Models were adjusted for infant sex, urbanization levels, pregnant age, preterm labor, low birth weight, gestational diabetes mellitus, and preeclampsia*.

**Table 4:** Overall risk of childhood allergic diseases in mothers with one, two, or three allergic diseases.

**Maternal status**	**Children allergic disease outcome**			
	**Overall**	**Asthma**	**AR**	**AD**
	**OR (95% CI)**	**OR (95% CI)**	**OR (95% CI)**	**OR (95% CI)**
**No. of allergic disease**				
0	1	1	1	1
1	1.52 (1.50–1.55)	1.30 (1.28–1.32)	1.54 (1.52–1.57)	1.33 (1.31–1.35)
2	1.93 (1.85–2.02)	1.89 (1.81–1.97)	1.86 (1.79–1.94)	1.65 (1.58–1.73)
3	2.73 (2.16–3.45)	1.96 (1.59–2.42)	2.30 (1.87–2.82)	2.44 (1.99–2.99)

*OR, odds ratio; AR, allergic rhinitis; AD, atopic dermatitis. The models were adjusted for infant sex, urbanization levels, pregnant age, preterm labor, low birth weight, gestational diabetes mellitus, and preeclampsia*.

## Discussion

In the present study, the risk of childhood allergic diseases, including asthma, AR, and AD, was associated with the maternal condition of pregnancy and autoimmune diseases. The following maternal pregnant conditions were associated with the risk of childhood asthma: high urbanization levels, preterm birth, young pregnancy age (<35 years), and low birth weight of infants. The risk ratio of childhood asthma increased among mothers with different IMIDs, including SLE, inflammatory myositis, and RA. The risk of childhood allergic disease significantly increased in maternal asthma, AR, and AD. The development of childhood asthma or allergic diseases is linked to environmental factors, including microbiomes, allergens, and air pollution (18). Our study showed that maternal conditions were related to the prevalence of early childhood asthma. The relationship between maternal IMIDs (SLE, RA, inflammatory myositis) and childhood asthma prevalence was significant. Furthermore, the pregnancy conditions of mothers were significant in the risk of childhood asthma. These conditions included urbanization level, preterm birth, and low birth weight.

Asthma is a chronic inflammatory disease of the pulmonary system involving large and small airways. Numerous inflammation-related cells, including neutrophils, eosinophils, basophils, and T lymphocytes, may infiltrate the narrowing airway (19). In the development of asthma, the progressive remodeling of the airway occurs through various inflammatory cells. Innate and adaptive immune systems are implicated in the disease progression of asthma. The systemic inflammation of the respiratory airway may be triggered by allergic exposure or other pathogens. Autoreactive or autoimmune responses to the dysregulation of the immune system occur (20). Genetic predisposition, environmental factors, and immune regulation play a major role in the pathogenesis of asthma or allergic diseases. Environmental factors include allergy, virus, bacteria, fungi, and pollutant materials. Air pollutants can also trigger the development of asthma or allergic diseases (21). Maternal exposure to air pollution during pregnancy increases the risk of childhood asthma (22). Fetal immune system workup in allergy is linked to maternal inflammations (23). Therefore, the status of the maternal immune system is associated with the development of childhood asthma. Our study showed an increased risk of childhood asthma in the following maternal status: pregnancy age of <35 years, preterm birth, and urban living. A population-based study in Finland revealed that the risk of childhood asthma diagnosed before the age of 3 years is associated with several maternal conditions, including maternal asthma, younger age, smoking, previous miscarriages, high number of previous deliveries, cesarean section, low gestational age, and low ponderal index (15). Regarding childhood asthma diagnosed at the age of 3 years or older, this Finnish study also found that maternal asthma is the strongest predictor; furthermore, young maternal age, low number of previous deliveries, short gestational age, and emergency cesarean section are related to an increased risk of asthma (15). In our study, high parity in mothers was associated with a decreased risk of childhood asthma. A previous study also indicated that a high number of siblings is associated with a decreased risk of atopic eczema, asthma wheezing, hay fever, and allergic sensitization (24). The risk of childhood asthma in infants living in the highest urbanization level was higher than that in infants residing in the lowest urbanization level (OR, 1.26; 95% CI, 1.24–1.29; *p* < 0.0001). In a previous study, the risk of asthma or AR is higher in children living in rural areas than in urban areas (25). The lower prevalence of childhood asthma in children living in farms is associated with asthma-protective “farm effect” (26). These environmental protective effects occur through innate immune signaling to inhibit airway hyperreactivity and eosinophilia (27). However, our study did not directly investigate the influence of the environmental factors of mothers or children on the development of asthma.

Autoimmune disease and allergy are involved in the dysregulation of the immune system. Numerous systemic inflammation-related cells and cytokine presentation are observed in this process. An allergic reaction triggers the imbalance of immune responses, including mast cells, eosinophils, neutrophils, and lymphocytes. During pregnancy, the maternal ratios of IFN-γ/IL-13 and IFN-γ/IL-4 are associated with a lower prevalence of childhood asthma (28). In a previous study, the increased prevalence of allergic rhinitis/conjunctivitis/asthma is associated with various IMIDs, including SLE, Sjögren's syndrome, myasthenia, vitiligo, and psoriasis (29). In our study, the mothers with IMIDs were diagnosed before pregnancy, and they were considered to have IMIDs if they had at least three ambulatory visits or one inpatient visit with a diagnosis of IMIDs. Our study showed an increased risk of childhood asthma in children born to mothers with maternal autoimmune diseases, including SLE, RA, and inflammatory myositis.

SLE is a systemic inflammatory disease with multi-organ involvement. Reactive autoantibodies are produced, and cytokines are presented during the disease course of SLE. Maternal SLE can influence the cytokine presentation of a fetus. Harmful infant outcomes, including preterm birth, infection, and mortality, are more common in SLE pregnancies (30). An increased risk of allergic diseases is observed in children whose mothers have SLE (10). Maternal SLE during pregnancy is associated with an increased risk of childhood asthma possibly because of preterm birth (9). Therefore, the risk of childhood asthma in children with mothers who have maternal SLE was higher in our study (OR, 1.13; 95% CI, 1.00–1.27).

Inflammatory myositis (dermatomyositis and polymyositis) is a chronic autoimmune disease involving muscular and integumentary systems. Moreover, the amount of circulating autoantibodies with increasing inflammatory cytokines increases in patients with inflammatory myositis. Our study revealed that maternal inflammatory myositis was related to an increased risk of childhood asthma (OR, 1.40; 95% CI, 1.12–1.74).

RA is another autoimmune disease with a clinical presentation of polyarthritis. Patients with RA have various autoantibodies. Predominant T-lymphocyte-related proinflammatory cytokines are significant in patients with RA. Protein kinases are activated by proinflammatory cytokines, and their activation can contribute to allergic or autoimmune diseases; furthermore, T lymphocytes play a major role in RA (31). Maternal RA is associated with an increased susceptibility of chronic diseases, including thyroid disease, epilepsy, type 1 diabetes, and asthma (32, 33). In our study, the ratio of childhood asthma was significantly higher in mothers with RA (OR, 1.21; 95% CI, 1.07–1.38).

The maternal history of asthma is significantly associated with childhood allergic diseases, including asthma, atopic dermatitis, and eczema (34). The risk of active childhood asthma is higher among mothers with asthma than among mothers without asthma (35). Genetic and epigenetic backgrounds are significant in the development of childhood asthma. Maternal asthma serves as a risk factor of childhood asthma because of maternal inflammation-related type-2 cytokines activating the fetal immune system during pregnancy (36). The risk of childhood asthma is higher in mothers with different pregnancy conditions, including asthma history, allergy, chronic bronchitis, and severe stress (37). In our study, maternal allergic disease was significantly related to childhood allergic disease (Table 3). A dose–response relationship was found in the relationship of the risk of childhood allergic disease with the number of maternal allergic diseases because of the compounding genetic and environmental effects (Table 4).

This study had some limitations. First, asthma had multiple contributing factors, including diet, infection, and air pollution, but they were not considered in this study. Second, childhood asthma at the age of 6 years without a follow-up beyond this age was evaluated.

In conclusion, this study revealed that childhood asthma was related to maternal status during pregnancy and maternal history of IMIDs or allergic diseases. The risk of childhood asthma at 6 years of age increased significantly in children whose mothers had IMIDs with SLE, RA, and inflammatory myositis. The higher prevalence of childhood allergic disease was linked to maternal allergic diseases, including asthma, AR, and AD. The risk of overall childhood allergic diseases increased with the number of maternal allergic diseases. The relationship between maternal IMIDs (SLE, RA, and inflammatory myositis) and childhood asthma prevalence was significant. Furthermore, pregnant mothers' conditions, including urbanization level, preterm birth, and low birth weight, were significantly associated with the risk of childhood asthma. Childhood asthma or allergic diseases were also related to genetic and non-genetic background. Our study elucidated the important role of genetic background in childhood asthma. Therefore, maternal immune and allergic conditions were linked to the progression of childhood allergic diseases. However, further, molecular or animal studies should be conducted to obtain additional data that could support this finding.

## Data Availability Statement

The data analyzed in this study is subject to the following licenses/restrictions: the original claim data were not allowed to carry out. Only analyzed tables or figures were allowed to carry out. Requests to access these datasets should be directed to Hsin-Hua Chen, shc5555@hotmail.com.

## Ethics Statement

The studies involving human participants were reviewed and approved by the Institutional Review Board of Taichung Veterans General Hospital. Written informed consent from the participants' legal guardian/next of kin was not required to participate in this study in accordance with the national legislation and the institutional requirements.

## Author Contributions

H-HC conceived and designed the study. D-HY and H-HC performed the literature search, interpretation of data, and drafted the manuscript. C-SC, W-CC, C-HL, and Y-WC conducted data extraction, methodological quality assessments, and performed the analysis. H-HC and Y-HC performed critical revision of the manuscript for important intellectual content. All authors read and approved the final version of submitted manuscript.

## Conflict of Interest

The authors declare that the research was conducted in the absence of any commercial or financial relationships that could be construed as a potential conflict of interest.

## Publisher's Note

All claims expressed in this article are solely those of the authors and do not necessarily represent those of their affiliated organizations, or those of the publisher, the editors and the reviewers. Any product that may be evaluated in this article, or claim that may be made by its manufacturer, is not guaranteed or endorsed by the publisher.
