# Supplementary material for: Association of the Risk of Childhood Asthma at Age 6 With Maternal Allergic or Immune-Mediated Inflammatory Diseases: A Nationwide Population-Based Study
Source: Front Med (Lausanne). 2021 Aug 2;8:713262. doi: 10.3389/fmed.2021.713262 (PMC8365169; doi:10.3389/fmed.2021.713262)
Supplement: Supplementary file 1 [file Table_1.DOCX]

Supplementary Table 1. Definitions of urbanization levels 1–7 in Taiwan

| Level | *Population density, persons/km^2^ | ^†^Inter-level ranking | *Percentage of education level ≥ college | ^†^Inter-level ranking | *Percentage of elderly (≥ 65 years) | ^†^Inter-level ranking | *Percentage of agriculture workers | ^†^Inter-level ranking | *Number of physicians, per 10^5^ people | ^†^Inter-level ranking |
| --- | --- | --- | --- | --- | --- | --- | --- | --- | --- | --- |
|  |  | ^‡^Intra-level ranking |  | ^‡^Intra-level ranking |  | ^‡^Intra-level ranking |  | ^‡^Intra-level ranking |  | ^‡^Intra-level ranking |
| 1 | 3.0863 | 1 | 1.4482 | 1 | -0.5993 | 5 | -1.0325 | 7 | 1.7014 | 1 |
|  |  | 1 |  | 3 |  | 4 |  | 5 |  | 2 |
| 2 | 0.3357 | 2 | 1.1896 | 2 | -0.8579 | 6 | -0.9623 | 6 | 0.6813 | 2 |
|  |  | 3 |  | 1 |  | 4 |  | 5 |  | 2 |
| 3 | -0.0883 | 3 | 0.1815 | 3 | -0.9706 | 7 | -0.7836 | 5 | -0.2546 | 4 |
|  |  | 2 |  | 1 |  | 5 |  | 4 |  | 3 |
| 4 | -0.3491 | 4 | 0.0001 | 4 | 0.0842 | 3 | -0.3273 | 4 | -0.1504 | 3 |
|  |  | 5 |  | 2 |  | 1 |  | 4 |  | 3 |
| 5 | -0.4365 | 6 | -0.3143 | 5 | 1.9476 | 1 | 0.8304 | 2 | -0.4250 | 7 |
|  |  | 5 |  | 3 |  | 1 |  | 2 |  | 4 |
| 6 | -0.4443 | 7 | -0.9060 | 7 | 0.7853 | 2 | 1.3880 | 1 | -0.3095 | 5 |
|  |  | 4 |  | 5 |  | 2 |  | 1 |  | 3 |
| 7 | -0.4053 | 5 | -0.8059 | 6 | -0.2210 | 4 | 0.6958 | 3 | -0.3338 | 6 |
|  |  | 4 |  | 5 |  | 2 |  | 1 |  | 3 |

*Data are shown as mean values of standardized score; formula of standardized score is: Xsd_i_ = (X_i_ – Mean of X_i_)/(Standard Deviation of X_i_); X_i_: the value of each index, i = 1,2,….,5

^†^Inter-level ranking: ranking among different levels

^‡^Intra-level ranking: raking among different variables
